# The number of granular cells in a cerebellar neuronal network model engaged during robot control increases with the complexity of the motor task

**DOI:** 10.1186/1471-2202-15-S1-P143

**Published:** 2014-07-21

**Authors:** Ruben-Dario Pinzon-Morales, Yutaka Hirata

**Affiliations:** 1 Department of Computer Science, Chubu University Graduate School of Engineering, Kasugai, 487-8501, Japan

## Introduction

The granular cells (Gr) in the cerebellum have been proposed to perform adaptive spatio-temporal coding [1] to relay rich information to the Purkinje cell, and thus they are critical for the proper operation of the cerebellum [2]. Elimination of all Gr cells induced behavioral symptoms such as ataxia and hypotonia [3]. Partially silencing Gr cells showed that the overall motor performance was minimally affected whereas demanding tasks or memory consolidation processes were compromised [4]. Here we use a physio-anatomically inspired cerebellar neuronal network (CNN) to study the role of the Gr cells during control of an unstable two-wheel balancing robot [5].

## Methods

The CNN comprises 755 Gr, 5 Golgi (Go), 15 basket/stellate (Ba/St), and 1 Purkinje (Pk) cells. Inputs to the CNN carried by mossy fibers (mfs) provided the desired motion trajectories of the robot. Inhibitory feedback loop between Gr and Go, and feed-forward inhibitory loop between Ba/St and Pk were included as in the real cerebellum. A proportional and differential (PD) controller sharing the same mfs inputs was introduced to represent the non-cerebellar input to the vestibular nucleus (Vn). The Vn computed the arithmetic difference between the PD and the Pk output and produced the motor command to drive the robot. The error signal to the Pk cell is conveyed by the climber fiber (cf). Bidirectional plasticity at Gr-Pk synapses (LTP/LTD) induced by the cf was implemented as a basis for motor learning [6]. The desired motion of the wheel angle position of the robot was sinusoidal waves or a band limited random noise (BLRN) while that of the body tilt angle was zero. The complexity of the control task changed from *simple* to *difficult* as the frequency of the sinusoidal wave changed form 0.1 Hz to 0.4 Hz, and *very difficult* when the BLRN (cutoff frequencies 0.2 Hz and 0.5 Hz) desired motion was used. The sinusoidal desired motion was given for 100 cycles and the BLRN for 10 s. At the end of the experiment the Gr-Pk synaptic weights were sorted to identify the number of cells that contributed to the 90% of the input to the Pk cell.

## Results

It was found that less than half of the Gr cells in the CNN were required to form 90% of the Pk output for all the desired motion employed. Interestingly, as the complexity of the motor task increased, the number of Gr cells required also increased. Knocking down the output of the most contributing 100 Gr cells showed that the motor performance was momentarily affected, but the CNN was able to re-adjust the other Gr-Pk synaptic weights and recover the motor performance. These results are in line with other computational models, such as [7] that predicted that motor performance would be sustained despite using only a portion of Gr cells.

## Conclusions

In our robot control framework, the number of Gr cells driving the Pk cell output increased with the complexity of the motor task. Sparse coding in the Gr layer permitted the engagement of Gr cells to foster the motor performance.
